# Combining Multiparametric Magnetic Resonance Imaging and Prostate-Specific Antigen Density in Selecting Patients for Prostate Biopsy

**DOI:** 10.7759/cureus.105201

**Published:** 2026-03-13

**Authors:** Goran Derimachkovski, Georgi Georgiev, Krastyo Draginov, Boris Petkov, Valentin Rumenov, Dimitar Stoilov, Yordan Tomov, Vasil Vasilev, Krassimir Yanev

**Affiliations:** 1 Urology, Multiprofile Hospital for Active Treatment "Saint Sofia", Sofia, BGR; 2 Urology, Hill Clinic, Sofia, BGR; 3 Urology, University Multiprofile Hospital for Active Treatment "Aleksandrovska", Sofia, BGR

**Keywords:** magnetic resonance imaging, outcome assessment, prostate biopsy, prostate cancer, prostate-specific antigen density

## Abstract

Introduction: Multiparametric magnetic resonance imaging (mpMRI) combined with prostate-specific antigen density (PSAd) has emerged as an effective risk-stratification tool for guiding prostate biopsy decisions. This study aimed to determine the optimal, clinically feasible biopsy strategy by combining the mpMRI Prostate Imaging-Reporting and Data System (PI-RADS) score and PSAd to minimize unnecessary biopsies while maximizing the detection of clinically significant prostate cancer (csPCa; Gleason score {GS} ≥7), using transperineal targeted and systematic biopsy as the reference standard.

Methods: We retrospectively analyzed data from 511 men with clinical suspicion of prostate cancer (PCa) (PSA: >4 ng/mL; PI-RADS ≥3), who underwent pre-biopsy mpMRI and a subsequent combination of targeted and systematic transperineal prostate biopsy. Various mpMRI/PSAd thresholds were assessed based on their accuracy for detecting csPCa, their predictive value, the proportion of avoided biopsies, and net benefit and outcomes from decision curve analysis.

Results: The overall analysis showed that the optimal strategy was to perform biopsy for lesions assessed as PI-RADS 4 and 5 or PI-RADS 3 lesions with PSAd of ≥0.15 ng/mL/mL. This combined approach yielded a negative predictive value (NPV) of 91% and a positive predictive value (PPV) of 43%. Implementing this strategy would have avoided biopsy in 16% (80/511) of the men and reduced the overdiagnosis of insignificant PCa by 9% (15/159) while missing only 4% (7/194) of csPCa cases.

Conclusion: Combining mpMRI with PSAd provides a robust, risk-stratified approach to PCa diagnosis. Men with equivocal mpMRI findings (PI-RADS 3) and low PSAd (<0.15 ng/mL/mL) may safely defer immediate prostate biopsy, reducing unnecessary procedures and the burden of overdiagnosis.

## Introduction

Prostate cancer (PCa) is the most frequently diagnosed malignancy in men in Europe and ranks as the third leading cause of cancer-related death in this population [1]. While younger men are more likely to die from PCa, older men often die with the disease rather than from it [2]. Importantly, PCa encompasses a spectrum from clinically significant disease, which requires active treatment, to clinically insignificant forms: low-volume, low-grade tumors that may never impact a patient's lifespan. The diagnosis and treatment of clinically insignificant prostate cancer (insPCa) can expose patients to unnecessary interventions with the risk of impairing the quality of life without conferring a survival benefit [3].

Historically, an elevated prostate-specific antigen (PSA) level or abnormal digital rectal examination was sufficient to justify a random prostate biopsy [4]. Given the potential complications associated with biopsies, such as bleeding, infection, and the resultant increase in healthcare costs, current European Association of Urology (EAU) guidelines now recommend performing multiparametric magnetic resonance imaging (mpMRI) as a triage tool in asymptomatic men with elevated PSA levels prior to prostate biopsy [5]. However, a notable limitation of mpMRI arises in the context of equivocal lesions, which are often considered positive by guidelines. Adopting a "biopsy all" approach for them can lead to a substantial number of unnecessary biopsy procedures.

This highlights the need for additional predictors to supplement mpMRI findings and help accurately distinguish men who require diagnostic biopsies from those who might safely avoid them. Prostate-specific antigen density (PSAd) has emerged as a promising adjunct [6]. While PSAd alone has limited utility for guiding biopsy decisions [6,7], its predictive value significantly improves when combined with mpMRI results, particularly in enhancing the negative predictive value for ruling out clinically significant prostate cancer (csPCa) [8-10].

Therefore, the objective of this study was to combine PSAd with the Prostate Imaging-Reporting and Data System (PI-RADS) score to identify the most clinically feasible biopsy strategy that minimizes unnecessary biopsies, reduces insPCa detection, and minimizes the number of missed csPCa cases. Transperineal systematic and targeted biopsy served as the reference standard.

## Materials and methods

**Figure 1:**
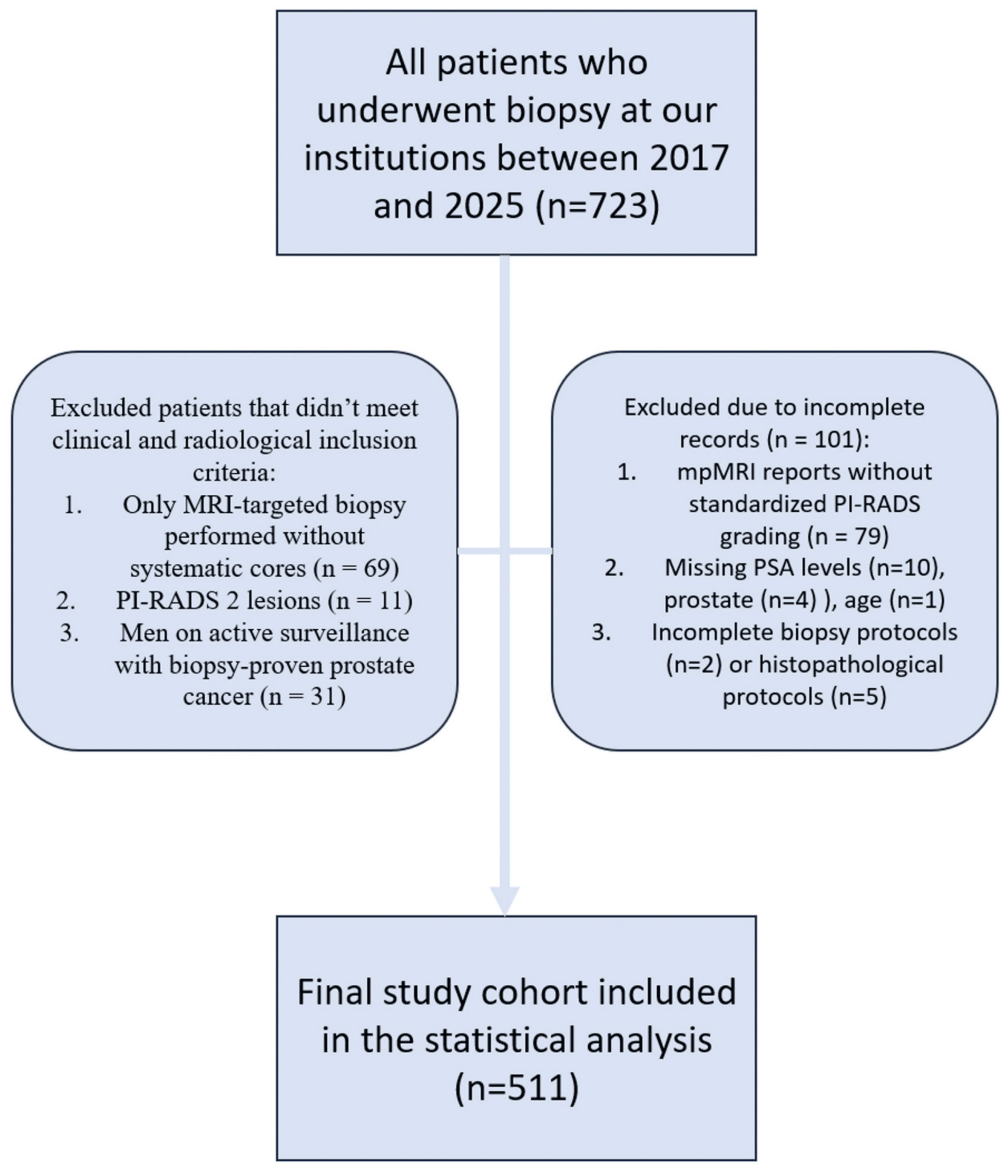
Flowchart of the patient selection process. The diagram illustrates the recruitment and exclusion pathway from the initial pool of 723 patients to the final cohort of 511 participants. Reasons for exclusion include methodological deviations, radiological inadequacy, and incomplete data records. MRI, magnetic resonance imaging; PI-RADS, Prostate Imaging-Reporting and Data System; mpMRI, multiparametric MRI; PSA, prostate-specific antigen

We retrospectively identified 723 consecutive sampled patients who underwent upfront prostate magnetic resonance imaging (MRI) and subsequent combined MRI-targeted and systematic biopsies at the Hill Clinic and Multiprofile Hospital for Active Treatment "Saint Sofia" in Sofia, Bulgaria, from the adoption of fusion biopsy technology in 2017 to 2025. Consistent with the national demographic profile, the population was racially homogeneous, consisting almost exclusively of Caucasian men. The inclusion criteria were defined to reflect the real-world clinical suspicion of prostate cancer: serum PSA levels elevated above 4 ng/mL, measured within six months prior to biopsy; the presence of at least one PI-RADS ≥3 lesions on MRI; and no prior diagnosis of PCa, either biopsy-naïve or with a previous negative biopsy. We specifically included only patients with PI-RADS ≥3 lesions because PI-RADS 1-2 cases often do not proceed to biopsy in clinical practice unless other high-risk indicators are present, and our goal was to evaluate how PSAd can further risk-stratify patients who already have suspicious findings on MRI. Patients with incomplete clinical, radiological, or histopathological data were excluded from the study. The flowchart of the patient selection process is illustrated in Figure 1. The final analysis included 511 patients, and their clinical characteristics are summarized in Table 1.

**Table 1:** Patients characteristics. Data are presented as median (interquartile range). ^a^Differences between groups of patients with csPCa and those without PCa or with insPCa were analyzed using one-way ANOVA^b^ for normally distributed data and the Kruskal-Wallis^c^ test for non-normal distribution. csPCa, clinically significant prostate cancer; insPCa, insignificant prostate cancer; IQR, interquartile range; PCa, prostate cancer; PSA, prostate-specific antigen; PSAd, prostate-specific antigen density

Clinical characteristics	no PCa (n = 158, 31%)	insPCa (n = 159, 31%)	csPCa (n = 194, 38%)	P-value^a^	Total (n = 511)
Age (year), median (IQR)	63 (57-68)	66 (62-72)	69 (63-74)	<0.001^b^	66 (61-71)
PSA (ng/mL), median (IQR)	7.89 (5.67-11.00)	9.00 (6.47-13.40)	11.55 (7.77-19.75)	<0.001^b^	9.20 (656-14.17)
Prostate volume (mL), median (IQR)	65 (50-90)	46 (32-63)	49 (37-65)	<0.001^b^	52 (38-78)
PSAd (ng/mL/mL), median (IQR)	0.11 (0.08-0.17)	0.21 (0.13-0.34)	0.25 (0.15-0.43)	<0.001^c^	0.18 (0.11-0.32)

All patients underwent mpMRI of the prostate with an intravenous contrast agent within six months before biopsy. The scans and interpretation were performed according to the European Society of Urogenital Radiology guidelines and were scored by radiologists using PI-RADS version 2.1 protocols [11]. The imaging protocol included axial and coronal T2-weighted images, axial diffusion-weighted imaging, and apparent diffusion coefficient maps. An mpMRI finding was categorized as suspicious if a PI-RADS 3, 4, or 5 lesion was present [11]. Prostate volume was determined using the MRI-based prolate ellipsoid formula (volume = length × width × height × π/6). This volume was used to calculate the PSAd by dividing the serum PSA level by the prostate volume.

Prostate biopsies were performed by dedicated urologists using a transperineal MRI/transrectal ultrasound fusion-guided biopsy approach. For each MRI-identified lesion, a minimum of three targeted cores were taken, along with a concomitant bilateral systematic sampling. The systematic biopsies were performed by the same operator without blinding to the MRI report. A spring-loaded biopsy gun with an 18-G needle was used for all procedures.

Biopsy specimens were evaluated by pathologists in accordance with the 2019 International Society of Urological Pathology recommendations [12]. For each core that was positive for PCa, the location, Gleason score (GS), Gleason grade group, and percentage of cancerous tissue were reported. Each patient's final GS was assigned based on the highest overall score derived from all systematic and targeted biopsy samples. csPCa was defined according to Epstein criteria as GS ≥7 or GS 6 with more than 50% of the core length involvement, while a Gleason score 6 disease with ≤50% core involvement was categorized as clinically insPCa [13].

Data were organized and managed using Microsoft Office 2019 (Microsoft Corp., Redmond, WA). Statistical analyses were conducted using IBM SPSS Statistics for Windows version 26.0 (IBM Corp., Armonk, NY; released 2019). Tables and figures were generated from the analyzed dataset using these software tools. Descriptive statistics were employed to summarize the patient cohort's characteristics. Differences between clinical groups were assessed using one-way ANOVA, Kruskal-Wallis, or chi-square tests. The diagnostic performance of parameters for predicting csPCa was evaluated via receiver operating characteristic (ROC) curve analysis, calculating the area under the curve (AUC) with 95% confidence intervals (CI). Optimal PSAd cutoffs were determined using Youden's J index. Sensitivity, specificity, positive predictive value (PPV), and negative predictive value (NPV) were calculated for various biopsy strategies. The clinical utility of these strategies was further assessed using decision curve analysis. A p-value of <0.05 was considered statistically significant. The research was approved by the Ethics Committee of Multiprofile Hospital for Active Treatment "Saint Sofia" (protocol number: 583; date: 29/12/2025) and was conducted in compliance with the Declaration of Helsinki. Informed consent was waived by the Ethics Committee due to the retrospective nature of the study.

## Results

**Figure 2:**
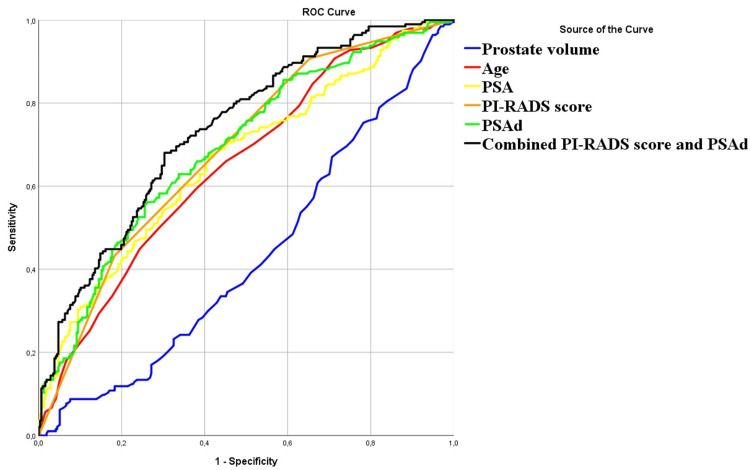
Predictive power of various individual detection measures for detecting clinically significant prostate carcinoma. ROC curves for prostate volume (AUC, 0.57; 95% CI, 0.52-0.62), age (AUC, 0.65; 95% CI, 0.60-0.70), PSA level (AUC, 0.66; 95% CI, 0.62-0.71), PI-RADS score (AUC, 0.68; 95% CI, 0.64-0.73), PSAd (AUC, 0.69; 95% CI, 0.65-0.74), and combination of PSAd and PI-RADS score (AUC, 0.74; 95% CI, 0.69-0.78). Diagonal segments are produced by ties. AUC, area under the curve; CI, confidence interval; PI-RADS, Prostate Imaging-Reporting and Data System; PSA, prostate-specific antigen; PSAd, prostate-specific antigen density; ROC, receiver operating characteristic

The final study cohort comprised 511 patients. Their clinical characteristics are presented in Table 1, showing distinct differences in age, PSA, prostate volume, and PSAd across patients without PCa, those with insPCa, and those with csPCa. Consistent with prior research, men with PCa tended to be older, with higher PSA levels, elevated PSAd, and smaller prostate volumes compared to those without PCa (Figure 2) [14,15]. We assessed the predictive value of individual parameters for csPCa detection using ROC curves. The combination of mpMRI and PSAd yielded the highest AUC of 0.74, indicating superior predictive performance over individual parameters.

PSAd significantly influenced the detection rate of PCa, particularly for PI-RADS 3 lesions. The overall PPV for csPCa in PI-RADS 3 lesions was 0.14. However, when applying PSAd thresholds, the PPV increased to 0.18 for PSAd of ≥0.1 ng/mL/mL, 0.23 for PSAd of ≥0.15 ng/mL/mL, and 0.19 for PSAd of ≥0.2 ng/mL/mL. Conversely, regarding NPV for csPCa in PI-RADS 3 score, a PSAd threshold of <0.10 ng/mL/mL yielded an NPV of 0.93, which decreased to 0.91 with PSAd of <0.15 ng/mL/mL and 0.88 with PSAd of <0.20 ng/mL/mL.

Overall, PCa was detected in 353 out of 511 patients (69%), with csPCa identified in 194 out of 511 patients (38%). The incidence of csPCa detected on biopsy progressively increased with rising MRI suspicion scores: 14% (18/128) for PI-RADS 3, 38% (92/241) for PI-RADS 4, and 59% (84/142) for PI-RADS 5. Concurrently, the proportion of men without PCa decreased from 60%, to 26%, to 13% across these categories, while the proportion of men with insPCa remained relatively consistent across the PI-RADS categories: 26%, 36%, and 28%, respectively.

**Figure 3:**
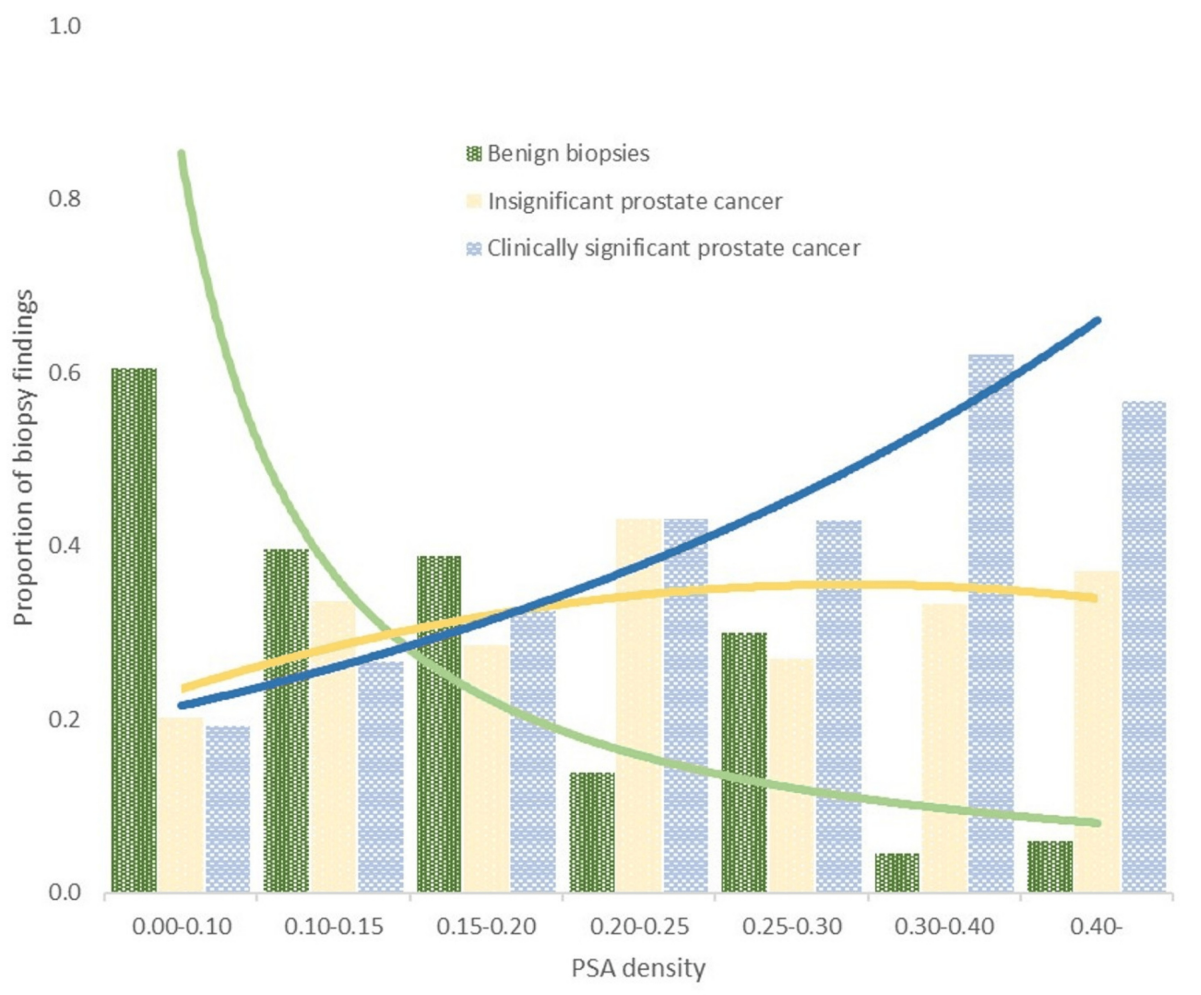
Distribution and predicted proportions of PCa findings stratified by PSAd thresholds. Each bar represents the proportion of patients without PCa, with insignificant PCa, and with clinically significant PCa within specific subgroups. The chart illustrates the increasing prevalence of clinically significant PCa in correlation with higher PSAd values across the study population. PCa, prostate cancer; PSAd, prostate-specific antigen density; PSA, prostate-specific antigen

The distribution of the 194 patients with csPCa across PSAd groups was broadly consistent for PSAd values up to 0.20 ng/mL/mL: 11% (21/194) had PSAd of <0.10 ng/mL/mL, 13% (26/194) had PSAd of 0.10-0.14 ng/mL/mL, and 13% (26/194) had PSAd of 0.15-0.19 ng/mL/mL. The majority (63%, 122/194) had PSAd of >0.20 ng/mL/mL. The risk of being diagnosed with csPCa directly correlated with increasing PSAd. The overall risk of csPCa was 40%. Specifically, the risk was 19% for men with PSAd of <0.1 ng/mL/mL, increasing to 27% for PSAd of 0.10-0.14 ng/mL/mL, 33% for PSAd of 0.15-0.19 ng/mL/mL, and 53% for PSAd of >0.2 ng/mL/mL. The proportion of men with GS 6 was relatively similar (29%-36%) for PSAd of >0.10 ng/mL/mL (Figure 3).

The prevalence of csPCa demonstrated a strong positive correlation with PSAd thresholds across all radiological categories. As detailed in Table 2, patients with PI-RADS 3 lesions exhibited a substantial increase in malignancy risk when PSAd exceeded 0.15 ng/mL/mL. Similar upward trends were observed in PI-RADS 4 and 5 categories, where higher PSAd levels consistently amplified the clinical suspicion of significant disease. This incremental risk pattern suggests that PSAd serves as a critical stratifying factor, particularly for equivocal mpMRI findings.

**Table 2:** Risk data table of csPCa, related to PI-RADS score and PSAd categories. Data are expressed as % (n/N), where N represents the total number of patients within the corresponding PI-RADS category and PSAd range, n denotes the number of patients diagnosed with clinically significant prostate cancer within N, and the percentage reflects the raw prevalence of csPCa for that specific subgroup. Data represent descriptive prevalence rates of csPCa prevalence within each subcategory. The risk of having csPCa is defined as very low if it is 0%-5% (below-population risk), low if 5%-10% (acceptable risk), intermediate-low if 10%-20%, intermediate-high if 20%-30%, high if 30%-40%, and very high if >40%. csPCa, clinically significant prostate cancer; PI-RADS, Prostate Imaging-Reporting and Data System; PSAd, prostate-specific antigen density

PI-RADS risk category	PSAd risk groups			
	Low	Intermediate-low	Intermediate-high	High
	<0.10	0.10-0.15	0.15-0.20	≥0.20
PI-RADS 3	6.5% (3/46)	11.8% (4/34)	29.4% (5/17)	19.4% (6/31)
PI-RADS 4	30.4% (14/46)	26.7% (12/45)	31.1% (14/45)	49.5% (52/105)
PI-RADS 5	18.8% (3/16)	55.6% (10/18)	40% (6/15)	68.8% (64/93)

Table 3 presents the diagnostic outcomes of integrating mpMRI scores and PSAd to evaluate various biopsy strategies and thresholds. The highest Youden's J index (0.26) was observed for patients with PI-RADS 4-5 lesions. Based on PPV and NPV, the most effective strategies were biopsy PI-RADS 4-5 or PI-RADS 3 with PSAd of ≥0.10 ng/mL/mL and biopsy PI-RADS 4-5 or PI-RADS 3 with PSAd of ≥0.15 ng/mL/mL. The first strategy showed a higher NPV, suggesting a better biopsy avoidance. Conversely, the second strategy demonstrated a higher PPV, indicating a greater likelihood of detecting csPCa. The second strategy also yielded a higher Youden's J index compared to the first.

**Table 3:** Results for different biopsy approaches with their corresponding sensitivity, specificity, and predictive values, presented with 95% confidence intervals, for detecting and ruling out significant prostate cancer when mpMRI PI-RADS categories are combined with various PSAd thresholds. CI, confidence interval; csPCa, clinically significant prostate cancer; insPCa, insignificant prostate cancer; mpMRI, multiparametric magnetic resonance imaging; NPV, negative predictive value; PI-RADS, Prostate Imaging-Reporting and Data System; PPV, positive predictive value; PSAd, prostate-specific antigen density

Biopsy restriction	Biopsies, n (%)		insPCa, n (%)		csPCa, n (%)		Diagnostic evaluation for csPCa				
	Performed	Avoided	Detected	Avoided	Detected	Missed	Sensitivity (95% CI)	Specificity (95% CI)	PPV (95% CI)	NPV (95% CI)	Youden's index
All men	511	0	159	0	194	0	Reference	Reference	Reference	Reference	
PI-RADS 4-5 or PI-RADS 3											
With PSAd of ≥0.10	465 (91)	46 (9)	152 (96)	7 (4)	191 (98)	3 (2)	0.99 (0.96-1.00)	0.13 (0.10-0.17)	0.41 (0.37-0.46)	0.93 (0.84-0.98)	0.12
With PSAd of ≥0.15	431 (84)	80 (16)	144 (91)	15 (9)	187 (96)	7 (4)	0.96 (0.93-0.98)	0.23 (0.19-0.28)	0.43 (0.39-0.48)	0.91 (0.84-0.96)	0.19
With PSAd of ≥0.20	414 (81)	97 (19)	140 (88)	19 (12)	182 (94)	12 (6)	0.94 (0.90-0.97)	0.27 (0.22-0.32)	0.44 (0.39-0.49)	0.88 (0.80-0.93)	0.21
PI-RADS 4-5											
	383 (75)	128 (25)	126 (79)	33 (21)	176 (91)	18 (9)	0.91 (0.86-0.94)	0.35 (0.30-0.40)	0.46 (0.41-0.51)	0.86 (0.79-0.91)	0.26
PI-RADS 5											
	142 (28)	369 (72)	40 (25)	119 (75)	84 (43)	110 (57)	0.43 (0.36-0.50)	0.82 (0.77-0.86)	0.59 (0.51-0.67)	0.70 (0.65-0.75)	0.25

**Figure 4:**
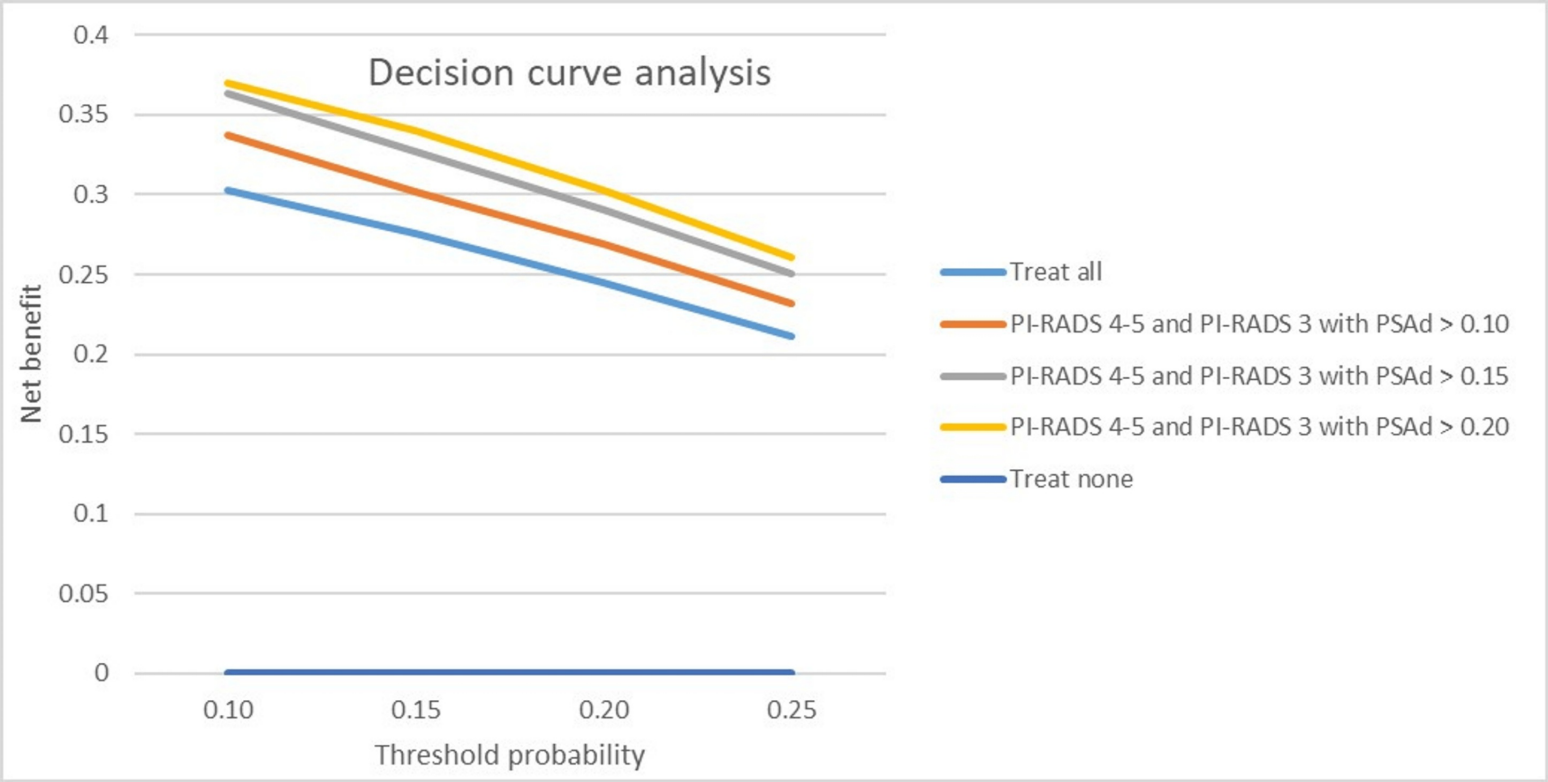
Decision curve analysis comparing the clinical utility of different biopsy strategies for detecting clinically significant prostate cancer using threshold probabilities ranging from 10% to 25%, which is equivalent to performing biopsies in 10 men and in four men, respectively, to find one man with clinically significant prostate cancer. The reference strategy was to biopsy all men. The biopsy strategy with the highest net benefit at a specific threshold probability had the greatest clinical value. PI-RADS, Prostate Imaging-Reporting and Data System; PSAd, prostate-specific antigen density

A decision curve analysis was performed using the strategies outlined in Table 3 (Figure 4). The analysis revealed that the diagnostic strategies offering the highest net benefit, in terms of biopsy avoidance and csPCa detection, were as follows: to biopsy PI-RADS 4 and 5 lesions and PI-RADS 3 lesions if PSAd is ≥0.2 ng/mL/mL, which reduced biopsies by 19% (97/511) and the overdiagnosis of insignificant cancers by 12% (19/159) while missing only 6% (12/194) of men with csPCa, or to biopsy PI-RADS 4 and 5 lesions and PI-RADS 3 lesions if PSAd is ≥0.15 ng/mL/mL, resulting in biopsy avoidance in 16% (80/511) of cases and a 9% (15/159) reduction in insPCa diagnoses while missing 6% (7/194) of csPCa cases. According to Youden's Index, the optimal PSAd thresholds were ≥0.20 ng/mL/mL for PI-RADS 3 lesions and for the overall PI-RADS 4 and PI-RADS 5 groups. The highest NPV for PI-RADS 3-5 lesions on mpMRI (considered positive) was observed for patients with PI-RADS 4-5 or PI-RADS 3 with PSAd of <0.10 ng/mL/mL (93%), while the highest PPV was achieved for patients with PI-RADS 4-5 or PI-RADS 3 with PSAd of ≥0.20 ng/mL/mL (44%). If a PSAd value of 0.15 ng/mL/mL had been adopted as the threshold to defer a biopsy in men with equivocal MRI results, 63% of biopsies could have been avoided.

## Discussion

The role of mpMRI in PCa diagnosis has been extensively studied, with two key trials, PRECISION [16] and PROMIS [17], providing level 1 evidence. These trials demonstrated that using mpMRI as a triage tool can reduce the need for prostate biopsies in one of four men while also improving the detection ratio of csPCa to insPCa. In addition to mpMRI suspicion scores, numerous studies have highlighted the significant influence of PSAd on the predictive values for both detecting and ruling out GS ≥7 PCa [8-10]. The biological rationale for utilizing PSAd over absolute PSA values lies in its ability to account for the confounding effect of benign prostatic hyperplasia. By normalizing the PSA level to the volume of the prostate gland, PSAd provides a more accurate reflection of the "excess" PSA production that is likely attributable to high-grade cancer cells rather than benign stromal or epithelial expansion [5].

Our findings confirm that the previously reported association between PSAd and the risk of harboring PCa is largely confined to high-grade tumors (GS ≥7), whereas the proportion of GS ≤6 cancers is consistent across different PSAd strata [7,18]. This supports the notion that high-grade tumors have an increased PSA diffusion into the circulation, while PSA passage in low-grade tumors is comparable to that in benign prostatic tissue.

We strived to define biopsy decisions based on mpMRI and PSAd combined. We found that in the current EAU recommendations for negative MRI results (PI-RADS ≤2) and high clinical suspicion, particularly when PSAd is ≥0.20 ng/mL/mL, systematic biopsy should be strongly considered due to the intermediate-to-high risk of csPCa in such patients [5].

However, for PI-RADS 3 lesions, the decision to biopsy is not so straightforward due to their equivocal nature and susceptibility to interobserver variability, particularly among general radiologists or those with a lower volume of prostate MRI interpretations (<3-5 years of experience) [19]. Several studies have explored strategies to refine biopsy decisions for these lesions [15,20]. While targeted biopsy is often the primary strategy, an alternative approach is to monitor lesion characteristics with subsequent MRI examinations. A study by Boschheidgen et al. demonstrated that patients with equivocal lesions who were later confirmed to have PCa typically exhibit an increase in PI-RADS score within 12-24 months, whereas those without PCa often show downgrading after 25-36 months [21].

Risk stratification by PSAd provides additional guidance: men with PI-RADS 3 lesions and low-risk PSAD (<0.10 ng/mL/mL) have a low probability (0%-6%) of csPCa, suggesting that biopsy could potentially be avoided. Conversely, a high-risk PSAd (>0.20 ng/mL/mL) is associated with a 15%-50% risk, warranting both targeted and systematic biopsy [22,23].

For positive mpMRI (PI-RADS 4-5), while PSAd does influence the PPV, the risk of csPCa is high enough to justify a biopsy regardless of the PSAd value [5].

While mpMRI and PSAd are valuable risk assessment tools for avoiding unnecessary biopsies, they cannot rule out any PCa. However, because most GS 6 cancers are considered indolent with minimal metastatic potential, the main clinical goal is to detect and rule out clinically significant disease [24].

Restricting biopsies to men with PI-RADS ≥4 or PI-RADS 3 with PSAd of ≥0.15 ng/mL/mL could have spared 16% (80/511) of men from biopsy, missing csPCa in only 9% (7/80) of these cases. Furthermore, diagnoses of insPCa, which can often be managed expectantly, were reduced by 9% (15/159), thereby lowering the burden of active surveillance programs.

Whether a 9% risk of missing a csPCa is acceptable is a matter of debate and should be a shared decision between the clinician and patient, carefully weighing the benefit of avoiding biopsy-related morbidity against the risk of missing a localized malignancy that could progress if left undetected. Most urologists would, however, not recommend a prostate biopsy for men with a PSA of up to 4 ng/mL and a clinically benign prostate, despite a similar prevalence of GS ≥7 cancer. For perspective, in the Prostate Cancer Prevention Trial [25], sextant biopsies of men with PSA of ≤4 ng/mL revealed GS ≥7 cancer in 5% of cases, though this proportion would likely be considerably higher today given the current biopsy protocols and updated grading criteria [26,27]. Similarly, autopsy studies have identified GS ≥7 cancer in 9% of men who died from unrelated causes [28].

Drawing from these and other trials, the EAU defines a patient with up to a 5% chance of having csPCa as being at a below-population risk and a patient with a 5%-10% chance as being at an acceptable risk, neither of which necessitates an immediate biopsy [5]. Our results align with these classifications: patients with a PI-RADS 3 lesion and PSAd of <0.10 ng/mL/mL had a 6.7% risk of csPCa, while patients with a PI-RADS 3 lesion and a PSAd between 0.10 and 0.15 ng/mL/mL had an 11.4% risk, which is close to the acceptable threshold. These findings are consistent with a similar study by Boesen et al., which reported risks of 6% and 8% for the same patient groups [22].

Our findings are concordant with several other studies that have examined the predictive value of PSAd as an adjunct to MRI results [8,10,14,22,29].

Boesen et al. conducted a prospective study of 808 men undergoing biparametric MRI with subsequent targeted and saturation biopsy irrespective of MRI findings, in which the optimal biopsy strategy was to restrict biopsies to men with MRI scores of ≥4 or scores of 1-3 with PSAd of ≥0.15 ng/mL/mL [22]. This approach yielded an NPV of 95% and a PPV of 56% for csPCa, potentially avoiding 41% of biopsies and 45% of overdiagnosed insignificant cancers, while missing 5% of csPCa. Our study, despite not including patients with PI-RADS 1-2 scores, applying Boesen's strategy, showed similar results with an NPV of 91% and a PPV of 43%, an avoidance of 16% of biopsies, and a missed csPCa rate of 4%. This suggests that PI-RADS 1-2 patients contribute significantly to the number of avoidable biopsies, while the rate of missed csPCa remains low.

In a similar study, Knaapila et al. found that for them, the most suitable strategy was to biopsy all MRI score 4-5 lesions and PI-RADS 3 lesions with PSAd of >0.20 ng/mL/mL, which resulted in a 6% rate of undetected csPCa [23]. A more aggressive approach was proposed by Falagario et al.: to biopsy all patients with positive or equivocal MRI results and all others with PSAd of >0.15 ng/mL/mL [29]. This approach was chosen to prioritize minimizing the risk of missing csPCa over biopsy reduction, highlighting the individualized nature of the biopsy decision.

Other retrospective studies have been published on the diagnostic accuracy of combining MRI and PSAd. For example, Rais-Bahrami et al. demonstrated improved PCa detection when biparametric MRI was combined with PSA or PSAd data, compared to either modality alone [14]. Similarly, Venderink et al. demonstrated that for PI-RADS 3 lesions, when the PSAd is less than 0.15 ng/mL/mL, the rate of false negatives was only 6%, resulting in a high negative predictive value of 94% for csPCa [30].

We acknowledge several limitations to our study. First, its retrospective nature introduces a potential selection bias, as our cohort likely overrepresents patients at higher risk for PCa. Consequently, patients with low PSAd and other low-risk parameters may be underrepresented, potentially influencing biopsy outcomes. Second, the administration of fluoroquinolones prior to repeat PSA screening and mpMRI to rule out benign causes of elevated PSA, such as urinary tract infections and prostatitis, may limit the generalizability of our findings to clinical settings without this pre-screening protocol. Third, the interpretation of MRI scans was performed by multiple radiologists, primarily at outpatient centers, without a secondary central review. This introduces potential inter-reader variability, which is a known confounding factor that may influence the consistency of PI-RADS scoring and, consequently, the performance of the proposed PSAd-based stratification [19]. However, while a central review might have improved internal validity, the use of multiple readers from different clinical sites reflects real-world practice, making the results generally relevant to similar clinical settings. Fourth, the exclusion of MRI-negative patients (PI-RADS ≤2) prevents the direct estimation of the true NPV of mpMRI. Fifth, there is a lack of racial diversity in the patient cohort, which was predominantly Caucasian. While this reflects the local demographics of Southeastern Europe, the findings may not be fully generalizable to other racial groups, such as men of African descent, who are known to have a higher biological risk for aggressive prostate cancer. Finally, a fundamental limitation is the inherent uncertainty regarding the true PCa prevalence. Like all prostate biopsy studies, the definitive prevalence of the disease requires whole-mount histopathological assessment following radical prostatectomy for all participants, which is neither feasible nor ethical. Therefore, our conclusions are based on biopsy results, which are subject to sampling error.

In future research, it could be prioritized prospective, long-term longitudinal studies specifically targeting the most controversial subgroup: patients with PI-RADS 3 findings and PSAd of ≤0.15 ng/mL/mL. It is essential to monitor these patients over an extended period to evaluate PSA velocity and follow-up mpMRI changes as triggers for delayed biopsy. Such studies should track the proportion of patients who eventually undergo biopsy and the subsequent detection rate of both insPCa and csPCa. Determining the biopsy-free survival and the oncological safety of this conservative approach will be crucial in establishing definitive guidelines for prostate cancer diagnostics.

## Conclusions

Combining mpMRI with PSAd enhances the diagnostic accuracy and improves predictive values for detecting csPCa in biopsy-naïve men with clinical suspicion of localized PCa. This combined risk assessment tool allows for the individualization of prostate biopsy decisions, enabling patients and clinicians to weigh the acceptable risk of missing csPCa against the desire to avoid unnecessary procedures. The most effective strategy observed was to restrict biopsy to men with highly suspicious mpMRI scores (PI-RADS ≥4) or those with equivocal scores (PI-RADS 3) and PSAd of >0.15 ng/mL/mL. For patients with equivocal MRI scores and PSAd of ≤0.15 ng/mL/mL, surveillance could be an appropriate strategy, reducing the overdiagnosis of insPCa and sparing patients from unnecessary invasive procedures. This approach facilitates more precise, informed, and patient-centered clinical decisions.
